# Minimally invasive esophagectomy: a propensity score-matched analysis of semiprone versus prone position

**DOI:** 10.1007/s00464-017-5975-1

**Published:** 2017-12-05

**Authors:** Maarten F. J. Seesing, Lucas Goense, Jelle P. Ruurda, Misha D. P. Luyer, Grard A. P. Nieuwenhuijzen, Richard van Hillegersberg

**Affiliations:** 1 Department of Surgery, University Medical Center Utrecht, Heidelberglaan 100, 3584 CX Utrecht, The Netherlands; 2 Department of Surgery, Catharina Hospital Eindhoven, Eindhoven, The Netherlands; 3 Department of Surgery, University Medical Center Utrecht, Heidelberglaan 100, 3508 GA Utrecht, The Netherlands

**Keywords:** Minimally invasive esophagectomy, Prone position, Semiprone position

## Abstract

**Background:**

The preferred surgical approach for esophageal cancer is a minimally invasive transthoracic esophagectomy with a two-field lymph node dissection. The thoracoscopic phase may be performed either in prone- or in left lateral decubitus (LLD) position. Prone positioning has been associated with better pulmonary outcomes compared to LLD positioning; however, conversion to a classic thoracotomy is more difficult. The semiprone position has been proposed as an alternative approach.

**Methods:**

A retrospective review of a prospectively maintained database (2008–2014) was performed to compare postoperative complications, surgical radicality, and lymph node yield between patients who underwent three-stage minimally invasive transthoracic esophagectomy in either the prone or semiprone position. Comparative analyses were conducted before and after propensity score matching.

**Results:**

One hundred and twenty-one patients were included. In total, 82 patients underwent minimally invasive esophagectomy (MIE) in semiprone position and 39 patients in prone position. After propensity score matching, both groups consisted of 39 patients. The operative time in the semiprone group was longer (368 vs. 225 min, *P* < 0.001) and in this group the lymph node yield was significantly higher (16 (range 6–80) vs. 13 (range 3–33), *P* = 0.019). There were no statistically significant differences regarding radical resections, postoperative complications, and hospital stay.

**Conclusion:**

The use of semiprone positioning in MIE is safe, feasible, and at least comparable to MIE in prone position in terms of oncological clearance and postoperative complications.

Curative treatment for locoregional tumors of the esophagus or gastroesophageal junction consists of neoadjuvant chemo(radio)therapy followed by surgical resection with radical lymphadenectomy [1]. Minimally invasive esophagectomy (MIE) has been shown to reduce the trauma of surgery compared to an open (transthoracic) approach, resulting in a decreased morbidity and mortality after learning curve completion [2–4]. As a consequence, this procedure gained popularity in the last decade, especially in high volume centers [5]. The first thoracoscopic esophagectomy was performed in 1992 with the patient in left lateral decubitus (LLD) position [6]. To improve the exposure of the posterior mediastinum and obtain better ergonomic results, some authors suggested changing LLD to a prone position, introduced in 1994 [7]. Currently, some retrospective studies suggest that MIE in prone position results in a reduction of pulmonary complications, blood loss, and an increase in mediastinal lymph node yield compared to MIE in LLD position [2]. Furthermore, MIE in prone position is suggested to decrease workload and provide better ergonomic results [3]. On the other hand, conversion to a classic thoracotomy is probably more difficult in the prone position and special equipment and training is necessary to put the patient in prone position. The use of a modified semiprone position might overcome this problem while retaining the benefits of the prone position [4]. Therefore, this study aimed to compare three-stage thoracoscopic esophagectomy in the prone position to the semiprone position with regard to postoperative complications and oncological clearance.

## Materials and methods

### Patients

A retrospective review of a prospectively maintained database was performed to compare postoperative complications, surgical radicality, and lymph node yield between patients who underwent MIE in prone and semiprone position. To create a homogeneous cohort, all consecutive patients who underwent a three-stage MIE with a two-field lymphadenectomy, gastric conduit reconstruction, and a left cervical anastomosis, either with or without neoadjuvant chemoradiotherapy, between April 2008 and January 2014 in the Catharina Hospital Eindhoven and the University Medical Center Utrecht were included. All procedures were performed by dedicated upper gastrointestinal oncologic surgeons who had extensive experience in MIE in either prone or semiprone position. Patients who underwent an emergency esophagectomy were excluded. Institutional Review Board approval for both centers was obtained, and informed consent requirement was waived for this study.

### Anesthesia

**Fig. 1:**
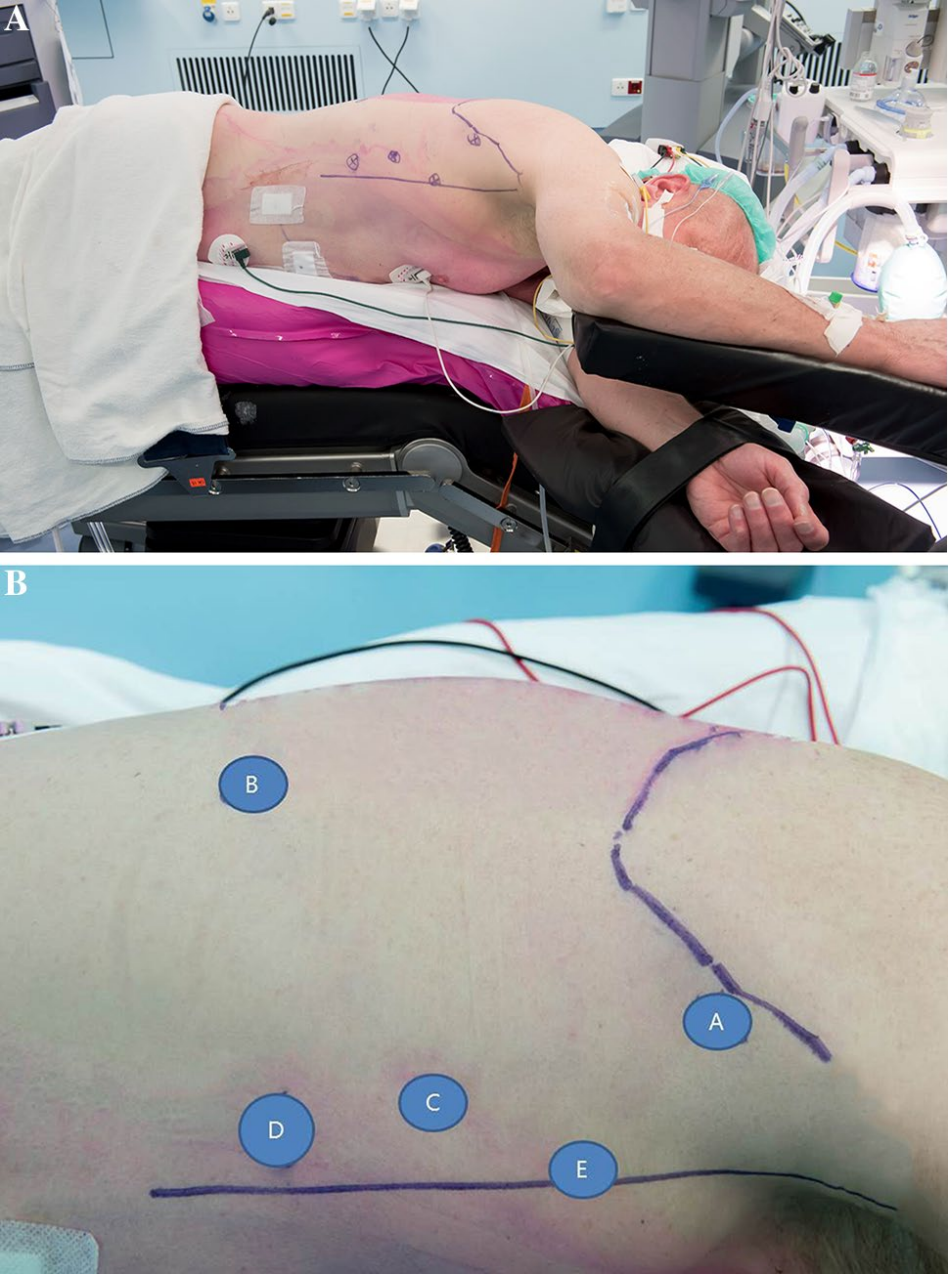
**A** Patient positioned in semiprone position. **B** Trocar placement in the semiprone position. Robotic arms 1 (*A*) and 2 (*B*) camera (*C*), and two assisting ports (*D*,* E*)

**Fig. 2:**
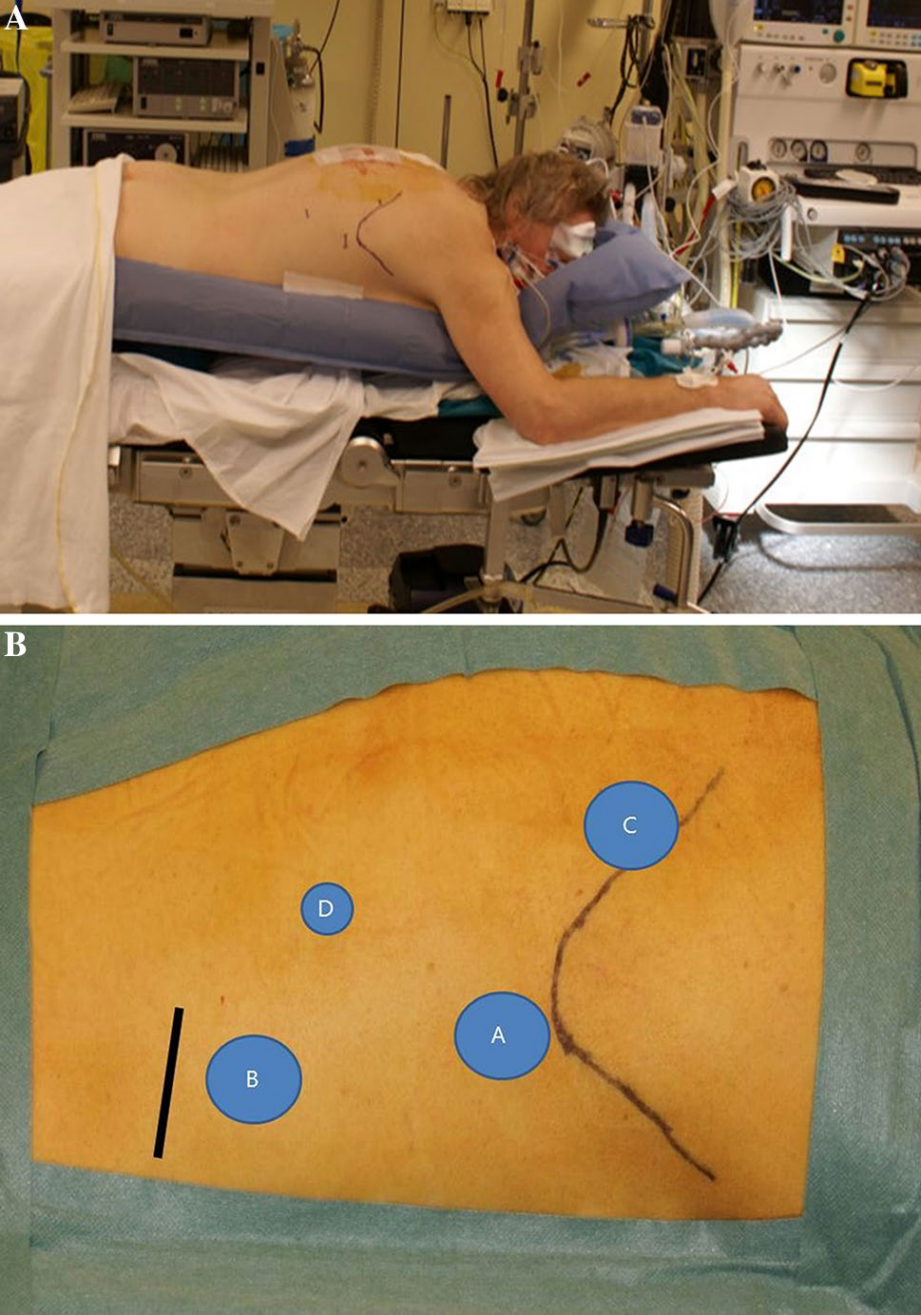
**A** Patient positioned in prone position. **B** Trocar placement in the prone position. A camera port (*A*), two 12-mm ports (*B*,* C*), and a 5-mm port (*D*)

In order to provide adequate analgesia, all patients received an epidural catheter (intercostal space T5-6, T6-7, or T7-T8) prior to surgery. Following prophylactic antibiotics (cefazolin 2.000 mg, metronidazole 500 mg), general anesthesia (intravenous propofol, sufentanil, and a muscle relaxant) were administered. In the semiprone group, endotracheal intubated was accomplished with a left-side double-lumen tube to enable desufflation of the right lung during the thoracic phase of the procedure. Subsequently, the patient was positioned in LLD position, tilted 45° to the prone position (Fig. 1A). A pressure-controlled ventilation strategy with a maximum pressure of 27 cmH_2_O and maximum tidal volume of 6 mL/kg was used during single-lung ventilation. To maintain end-tidal CO_2_ between 40 and 45 mmHg, tidal volumes were set at 6–8 mL/kg during double-lung ventilation. In the prone group, patients were placed in the swimmers position after intubation with a single-lumen endotracheal tube according the conventional procedure (Fig. 2A). In this group, during the thoracic phase, double-lung ventilation was maintained and an optimal view was established with 8 mmHg insufflational pressure. Tidal volumes were set between 3 and 5 mL/kg and ventilatory frequency between 18 and 28 per minute.

### Surgical technique

A three-stage esophagectomy was performed with cervical anastomosis (McKeown procedure). Transthoracic mobilization of the esophagus and mediastinal lymph nodes dissection was performed in either semiprone or prone position (see description below). Subsequently, the patient was placed in supine position for conventional laparoscopic mobilization of the stomach, truncal lymph node dissection, and an extracorporal gastric conduit formation. Finally, a left cervical esophagogastric anastomosis was created through a left vertical neck incision along the anterior border of the sternocleidomastoid muscle.

### Semiprone

Three ports were placed for the robotic system, as well as two ports for the assisting surgeon (Fig. 1B). After desufflating the right lung, CO_2_ at 6 mmHg was insufflated through one of the assistant ports to keep the lung out of the operative field. Following division of the right pulmonary ligament, an extensive mediastinal lymph node dissection was performed (para-esophageal, infracarinal, aortic pulmonary window, subcarinal, paratracheal). The azygos arch was ligated and the thoracic duct was clipped at the level of the diaphragm. Hereafter, the esophagus was mobilized and resected en bloc with the surrounding mediastinal and subcarinal lymph nodes, and the thoracic duct. This technique has been described more thoroughly in previous studies [8–10].

### Prone

A 12-mm camera port was placed posterior to the tip of the scapula and CO_2_ insufflation was accomplished with a pressure of 8 mmHg. Second and third 12-mm ports were placed in the eighth intercostal space in the right posterior axillary line and medial of the scapula, respectively. A 5-mm port was placed halfway between the spine and the original eighth intercostal port (Fig. 2B). By means of conventional thoracoscopy, the mediastinal pleura was opened with a harmonic scalpel with subsequent mobilization of the esophagus. A mediastinal lymph node dissection was performed (para-esophageal, infracarinal, aortic pulmonary window, subcarinal). A paratracheal lymph node dissection was not routinely performed and only on indication when lymph node metastases were suspected on preoperative staging. The azygos vein was clipped with a Hemolock^®^ and transected. The main bronchi, trachea, and pleura were dissected towards cranial. Hereafter, the pleura was dissected towards the diaphragm where the thoracic duct was clipped. The mobilized esophagus was resected en bloc with the mediastinal lymph nodes.

### Postoperative treatment

After surgery, patients were admitted to the intensive care unit (ICU), while maintaining mechanical ventilation. Extubation was performed when patients were considered cardiorespiratory stable. Epidural analgesia was maintained during the first postoperative day. In addition, all patients were provided with patient-controlled opioid analgesia.

### Outcomes

The primary outcome was pneumonia, which was defined according to the criteria of the Uniform Pneumonia Score [11]. Secondary outcomes included intra-operative variables, such as total blood loss, length of operation, and conversion rate. Furthermore, the number of harvested lymph nodes, radicality, in-hospital mortality, pulmonary embolism, clinically or radiologically proven anastomotic leakage, mediastinitis, chylothorax, recurrent laryngeal nerve palsy, wound infection, cardiac complications and ICU, and hospital stay were also included as secondary outcome measures.

### Statistical analysis

Perioperative outcomes of patients who underwent MIE were compared between patients in the semiprone and prone position. Propensity score matching was performed to create comparable treatment groups (prone versus semiprone) with regard to measured confounders. First, a propensity score for each patient was computed by logistic regression using the position of the patient during surgery as the dependent variable and the variables marked in Table 1 as covariates. Equal study groups were created by one-to-one nearest-neighbor matching without replacement. Values were presented as mean ± standard deviation (SD) or median (range). The demographic and clinical data of both surgical groups were compared with a Chi-square test or a Mann–Whitney *U* test. Statistical analyses were performed using SPSS version 23.0 (IBM Corp., Armonk, NY) and R 3.1.2 open-source software (http://www.R-project.org; ‘MatchIt’ and ‘optmatch’ packages). A *P* value of < 0.05 was considered statistically significant.

**Table 1:** Patient and treatment-related characteristics in relation to surgical procedure

Characteristic	Before matching					After matching				
	Semiprone (*n* = 82)		Prone (*n* = 39)		*P* value	Semiprone (*n* = 39)		Prone (*n* = 39)		*P* value
	*n*		*n*			*n*		*n*		
Gender										
Female	31	37.8%	10	26%	0.649	13	33%	10	26%	0.456
Male	51	62.2%	29	74%		26	67%	29	74%	
Age (years)	62	± 8.68	63	± 8.93	0.186	63	± 25.33	63	± 8.93	0.492
BMI (kg/m^2^)^a^	24.95	± 4.36	24.55	± 4.5	0.515	25.34	± 4.86	24.55	± 4.5	0.478
ASA score										
I	22	27%	1	2%	0.004	6	15%	1	2%	0.140
II	49	60%	28	72%		24	62%	28	72%	
III	11	13%	10	26%		9	23%	10	26%	
COPD										
No	69	84.1%	35	90%	0.408	33	85%	35	90%	0.498
Yes	13	15.9%	4	10%		6	15%	4	10%	
Cardiac comorbidity										
No	61	74.4%	34	87%	0.109	34	87%	34	87%	1.000
Yes	21	25.6%	5	13%		5	13%	5	13%	
Diabetes mellitus										
No	75	91.5%	35	90%	0.758	37	95%	35	90%	0.395
Yes	7	8.5%	4	10%		2	5%	4	10%	
Smoking										
No	29	35.4%	23	59%	< 0.001	19	49%	23	59%	0.364
Yes	53	64.6%	16	41%		20	51%	16	41%	
Alcohol										
No	25	30.5%	17	44%	0.174	13	33%	17	44%	0.532
Yes	53	64.6%	22	46%		26	67%	22	46%	
cT stage										
T1,2	24	29%	8	21%	0.307	10	26%	8	21%	0.591
T3,4	58	71%	31	79%		29	74%	31	79%	
cN stage										
N0	26	32%	8	21%	0.200	10	26%	8	21%	0.591
N+	56	58%	31	79%		29	74%	31	79%	
Histology										
ADC	43	53%	21	54%	0.938	23	59%	21	54%	0.648
SCC	39	47%	18	46%		16	41%	18	46%	
Tumor location										
Proximal	6	7%	0	0%	0.093	2	5%	0	0%	0.108
Middle	22	27%	16	41%		9	23%	16	41%	
Distal	54	66%	23	59%		28	72%	23	59%	
nCRT										
No	30	36.6%	3	8%	0.001	7	20%	3	8%	0.176
Yes	52	63.4%	36	92%		32	80%	36	92%	

Data are expressed as *N* (%) or mean ± SD. Neoadjuvant chemoradiotherapy consisted of intravenous carboplatin [AUC 2 mg/mL/min] and intravenous paclitaxel (50 mg/m^2^ of body-surface area) for 23 days with concurrent radiotherapy (41·4 Gy, given in 23 fractions of 1·8 Gy on 5 days/week)

*BMI* body mass index, *ASA* American Society of Anesthesiologists, *COPD* chronic obstructive pulmonary disease, *c* clinical, *nCRT* neoadjuvant chemoradiotherapy, *ADC* adenocarcinoma, *SCC* squamous cell carcinoma

## Results

A total of 121 patients were included. In 82 patients MIE was performed in semiprone position and in 39 patients in prone position. After propensity score matching both groups consisted of 39 patients. The clinical characteristics of the patients including propensity-matched groups are listed in Table 1. Patients in the prone group had a significantly higher ASA score, a smaller percentage of smokers, and a higher number of patients that received neoadjuvant chemoradiotherapy. All of these variables did not show statistically significant differences between the two groups after propensity score matching. Table 2 demonstrates the intra-operative outcome of the patients. There was no statistically significant difference in pneumonia rate in semiprone position (49%) versus prone position (36%), *P* = 0.252. The operative time in the semiprone group was longer (368 vs. 225 min, *P* < 0.001) and in this group the lymph node yield was significantly higher(16 (range 6–80) vs. 13 (range 3–33), *P* = 0,019). However, when all paratracheally harvested lymph nodes were excluded from analysis, this statistically significant difference ceases to exist (16 (range 2–78) vs. 13 (3–33), *P* = 0.128). There were no statistically significant differences between the semiprone and prone group in terms of blood loss (388 vs. 300 mL, *P* = 0.753), radical resections (both 92%, *P* = 0.946), and conversion rates [5 vs. 10%, *P* = 0.395 (total number of conversions) and 5 vs. 3%, *P* = 0.556 (conversions during thoracic phase)]. The incidence of in-hospital mortality (3 vs. 5%, *P* = 0.314), anastomotic leakage (26 vs. 36%, *P* = 0.326), mediastinitis (13 vs. 15%, *P* = 0.745), Chylothorax (38 vs. 28%, *P* = 0.337), recurrent laryngeal nerve palsy (10% vs. 8% *P* = 0.692), wound infection (15 vs. 26%, *P* = 0.644), cardiac complications (15 vs. 26%, *P* = 0.262) and the length of ICU (1 (range 1–16) vs. 1 day (range 1–30), *P* = 0.732), and total hospital stay (18 (range 8–87) vs. 17 days (range 7–84), *P* = 0.751) were not statistically different between the semiprone and prone group (Table 3).

**Table 2:** Surgical outcomes

	Before matching					After matching				
	Semiprone (*n* = 82)		Prone (*n* = 39)		*P* value	Semiprone (*n* = 39)		Prone (*n* = 39)		*P* value
	*n*		*n*			*n*		*n*		
Blood loss (mL)	320	(0–1460)	300	(100–680)	0.369	388	(197–547)	300	(100–6780)	0.753
Length of operation (min)	417	(318–547)	235	(147–336)	< 0.001	368	(50–1460)	225	(147–336)	< 0.001
Total lymph node yield	18	(5–80)	13	(3–33)	0.001	16	(6–80)	13	(3–33)	0.019*
R0 resection	77	94%	36	92%	0.685	36	92%	34	92%	0.946
Conversion (total)	7	9%	4	10%	0.896	2	5%	4	10%	0.395
Conversion (thoracic phase)	5	6%	1	3%	0.403	2	5%	1	3%	0.556

Data are expressed as *N* (%) or median (range)

*P = 0.128 after exclusion of paratracheally resected lymph nodes

**Table 3:** Postoperative outcomes

	Before matching					After matching				
	Semiprone (*n* = 82)		Prone (*n* = 39)		*P* value	Semiprone (*n* = 39)		Prone (*n* = 39)		*P* value
	*N*		*N*			*N*		*N*		
In-hospital mortality	5	6%	2	5%	0.831	1	3%	2	5%	0.556
Pneumonia	30	37%	14	36%	0.941	19	49%	14	36%	0.252
Pulmonary embolism	2	2%	0	0%	0.325	1	3%	0	0%	0.314
Anastomotic leakage	18	22%	14	36%	0.104	10	26%	14	36%	0.326
Mediastinitis	9	11%	6	15%	0.492	5	13%	6	15%	0.745
Chylothorax	30	37%	11	28%	0.363	15	38%	11	28%	0.337
Laryngeal nerve palsy/injury	6	7%	3	8%	0.941	4	10%	3	8%	0.692
Wound infection	3	4%	3	8%	0.339	2	5%	3	8%	0.644
Arrhythmia	14	17%	10	26%	0.269	6	15%	10	26%	0.262
Myocardial infarction	0	0%	0	0%	–	0	0%	0	0%	–
ICU stay	1	(1–35)	1	(1–30)	0.731	1	(1–16)	1	(1–30)	0.732
Hospital stay	16	(8–87)	17	(7–84)	0.879	18	(8–87)	17	(7–84)	0.751

Data are expressed as *N* (%) or median (range)

## Discussion

This study represents the first comparison between the semiprone and prone position for thoracoscopic mobilization of the esophagus. This study shows that the semiprone position is comparable to a prone position in terms of average estimated blood loss and postoperative complications such as pneumonia. Furthermore, the semiprone position is associated with an at least comparable lymph node yield and a similar percentage of radical resections. This illustrates that the semiprone position is safe and feasible in terms of postoperative complications and radical resections.

To date, few studies on positioning patients in semiprone position during MIE have been conducted, and most of them are small and descriptive of nature [12–14]. Studies comparing LLD with prone positioning are more common in literature. Recently, an extensive systematic review and pooled analysis on this subject found that prone positioning reduces pulmonary complications, blood loss, and increases mediastinal lymph node yield compared to the LLD position [11]. However, this comprehensive review also concluded that further studies are needed to explain performance-shaping factors and their influence on oncological clearance and short-term outcomes. Placing the patient in a semiprone position, hereby combining the benefits of the LLD and prone position, may be one of these performance-shaping factors.

This hypothesis is supported by the first and only comparative study on the feasibility and safety of placing the patient in semiprone position published to date [4]. This study, in which a comparison between the semiprone and LLD position was made, found no statistically significant differences in intra- and postoperative complications. Moreover, the semiprone position was associated with superior surgical ergonomics and better exposure of the posterior mediastinum. These findings, combined with the results of the current study, suggest that the profile of postoperative complications following MIE in the semiprone position is comparable to MIE in prone and LLD positioning.

No differences in conversion rates between groups were observed. Although it is a rare event, it has been suggested that if conversion to an open procedure is needed, a thoracotomy is most easily performed when the patient is already in LLD position. However, in the current study no severe difficulties were encountered during conversion to thoracotomy in either of the approaches. All conversions were performed due to reduced accessibility of the thorax because of adhesions, and no emergency conversion was needed. During MIE in semiprone position one can easily tilt the table to mimic the normal position to perform an open thoracotomy. When conversion to thoracotomy is needed during MIE in prone position, the patient needs to be repositioned and draped. However, since no emergency conversion was necessary in our series, we did not experience practical problems.

In the present study, MIE in the semiprone position required more trocar sites (5) than MIE in prone position (4), possibly leading to more surgical trauma for the patient. The semiprone position was associated with an increase in lymph node yield. However, since there are no statistically significant differences in lymph node yield between groups when all paratracheally harvested lymph nodes are excluded from analysis, this finding may rather be attributed to the decision, due to surgeons’ preference, of a more extensive paratracheal lymph node dissection in the semiprone position than to the position itself. The extensive paratracheal lymph node dissection, together with the set-up of the robotic platform may have led to a longer operative time. Nevertheless, the current study does show that the semiprone position, unlike the LLD position, does not have to come at the expense of the number of resected lymph nodes. This suggests that the effects of gravity in the prone position do not cease to exist after placing the patient in semiprone position, which reduces the need for lung retractions and gives better exposure of the posterior mediastinum, subcarinal, and paratracheal spaces, facilitating not only an extensive lymph node dissection but also the procedure as a whole [8]. Also the absence of deterioration of visibility due to the vertebral column in the semiprone position may further improve lymph node dissection.

There are several limitations to this study. First, all patients in the semiprone position were intubated with a left-side double-lumen tube, while patients in the prone position were ventilated through a single-lumen endotracheal tube. In both the prone and semiprone position, both the single-lumen and double-lumen can be used [15, 16]. The use of a double-lumen tube allows more controlled emptying of the lung on the operative side, prohibiting the lung to interfere with the operative field while the application of CPAP improves gas exchange, alveolar recruitment, and lung capacity [17].

On the other hand, ventilation through a single-lumen endotracheal tube has a distinct benefit in patients requiring prolonged ventilation post-operatively. This method does not require tube change at the conclusion of the procedure, preventing manipulation of the airway and therewith reduces the risk of airway loss and aspiration [18]. However, in the current era of enhanced recovery after surgery programs, most patients are extubated on the day of surgery and only few patients need prolonged ventilation after MIE, therefore this benefit of single-lumen tube ventilation is minor. The only studies published to date in which the use of a single-lumen tube is compared with the use of a double lumen in transthoracic MIE report no significant differences in postoperative (pulmonary) complications [19, 20]. Second, despite correction for baseline characteristics through propensity score matching, the inability of propensity score matching to adjust for unknown confounders that could explain some of our findings remains a limitation. Finally, all MIE in semiprone position or prone position were performed in either the University Medical Center Utrecht or the Catharina Hospital Eindhoven, respectively. Although both hospitals provided similar postoperative care, this might have introduced some bias. Nevertheless, inclusion of patients who are placed in either one of the positions in the same hospital would be very challenging, as in very few hospitals MIE is performed in both positions.

In conclusion, our findings suggest that the semiprone position during MIE is safe, feasible, and comparable to MIE in prone position in term of radicality and postoperative complications. Therefore, we would recommend to position the patient according to the surgeons preference.
